# Effects of Modest Carbohydrate–Energy Supplementation on Resistance Training Adaptations in Trained Men: A Crossover Trial

**DOI:** 10.3390/nu18121961

**Published:** 2026-06-17

**Authors:** Menno Henselmans, Dakota R. Tiede, Daniel L. Plotkin, Madison L. Mattingly, Emrie R. Harbour, Derick A. Anglin, Andrew D. Fruge, Fredrik Tonstad Vårvik, Michael D. Roberts, Mikel Izquierdo

**Affiliations:** 1 Navarrabiomed, Complejo Hospitalario de Navarra (CHN), Universidad Pública de Navarra (UPNA), 31008 Pamplona, Spain; 2 School of Kinesiology, Auburn University, Auburn, AL 36849, USA; 3 Department of Nutritional Sciences, Auburn University, Auburn, AL 36849, USA; 4 College of Nursing, Auburn University, Auburn, AL 36849, USA; 5 Department of Sport Science and Physical Education, University of Agder, 4630 Kristiansand, Norway; 6 Centro de Investigación Biomédica en Red de Fragilidad y Envejecimiento Saludable (CIBERFES), Instituto de Salud Carlos III, 28029 Madrid, Spain

**Keywords:** resistance training, carbohydrates, energy intake, muscle hypertrophy, strength

## Abstract

**Background/Objectives:** Higher energy and carbohydrate intakes have been hypothesized to enhance resistance training adaptations, yet empirical evidence remains mixed. The purpose of this study was to investigate whether supplemental carbohydrate–energy intake improves muscle hypertrophy and strength. **Methods:** Twenty resistance-trained men (26.7 ± 4.9 years old, 9.7 ± 6.1 years training experience) completed a quasi-randomized, double-blinded, counterbalanced crossover trial. Participants consumed either a daily protein-only supplement (30 g protein, 4 g carbohydrate) or a daily protein-plus-carbohydrate supplement (30 g protein, 54 g carbohydrate) for 8 weeks each, followed by crossover, while continuing their habitual training and nutrition. Primary outcomes included lean mass obtained using dual-energy X-ray absorptiometry, muscle thickness and cross-sectional area obtained via ultrasound, back squat one-repetition maximum, fatigue index, and knee extensor peak torque. Differences in estimated marginal means, controlling for order and phase effects, were analyzed via linear mixed models, with first-phase-only ANCOVAs as sensitivity analyses. **Results:** The carbohydrate–protein condition resulted in significantly higher daily energy (+485 kcal/d; *p* = 0.017) and carbohydrate intake (+33 g/d; *p* = 0.043) than the protein-only condition, with no differences in protein or fat intake or training volume. No significant differences between conditions were observed for any outcome, including in the sensitivity analyses. **Conclusions:** Modest supplemental carbohydrate–energy intake did not significantly augment muscle hypertrophy, fatigue resistance or strength in trained men within our study context. More high-powered research is needed to determine how much and under which circumstances carbohydrate–energy intake affects resistance training adaptations.

## 1. Introduction

Resistance-training-induced neuromuscular adaptations can improve health, physical performance, and sports performance [1]. Higher energy intakes could enhance these adaptations. Energy deficiency is an inherently catabolic stimulus that can reduce muscle anabolic signaling and protein synthesis (MPS) [2,3]; however, not all studies have observed decreased MPS as a result of energy restriction [4,5]. A 2021 meta-analysis by Murphy and Koehler [6] found that energy deficiency impaired resistance training-induced gains in fat-free mass but not strength. In contrast, outside of energy deficit, research findings on the effect of additional energy intake on resistance training-induced adaptations have been mixed. Rozenek et al. [7] found that two groups supplemented with 2010 kcal gained significantly more fat-free mass from resistance training than those on a habitual control diet. However, both groups had higher protein intakes than the control group, making it unclear whether the additional protein or energy intake stimulated the increase in fat-free mass. Moreover, strength gains did not differ significantly between the conditions, raising questions about whether the greater fat-free mass was contractile tissue. A four-week pilot study on 11 bodybuilders by Ribeiro et al. [8] found that a higher self-reported energy surplus improved muscle growth compared with a lower energy intake, as estimated using an anthropometric equation and skinfold calipers. In contrast, multiple studies have not observed significantly greater resistance training-induced neuromuscular adaptations resulting from increased energy intakes outside of energy deficit, even when the higher energy intakes resulted in significantly greater fat gain [9,10,11,12,13].

Mechanistically, little energy is required for muscle growth. Based on the metabolizable energy density of muscle tissue and the cost of building it, building even a kilo of muscle per month is estimated to require less than 100 kcal per day. This energy could be obtained by catabolizing fat mass, making it questionable whether an energy surplus is needed in the first place to maximize muscle growth [14].

Increased energy intake, particularly from additional carbohydrates, may enhance neuromuscular adaptations to resistance training. Carbohydrate intake has been hypothesized to enhance neuromuscular adaptations to resistance training, primarily because it can improve acute performance and support higher chronic training volumes [15,16]. However, empirical support for this argument is mixed. A meta-analysis by King et al. (2022) [17] found that pre-exercise carbohydrate ingestion could acutely enhance resistance training performance, yet the benefits were restricted to fasted-state comparisons, and no clear dose–response relationship was observed. Similarly, a systematic review by Henselmans et al. [18] found no ergogenic effect of carbohydrate supplementation in fed-state resistance training protocols involving ≤10 sets per muscle group, likely due to limited glycogen depletion. Conventional resistance training protocols typically result in only modest glycogen reductions (≤41% [19,20,21,22,23,24,25,26,27,28,29,30]), not reaching the critical glycogen storage threshold of 250–300 mmol/kg dry weight required to impair neuromuscular function [31]. For instance, Essén-Gustavsson and Tesch [26] observed 28% quadriceps glycogen depletion in bodybuilders after 20 sets of quadriceps training to failure. However, there is mixed evidence that type II muscle fibers specifically may experience greater depletion with resistance training volumes of ≥12 sets per muscle group per workout [21,27].

Longitudinal trials have generally failed to find an effect of carbohydrate consumption on resistance-training-induced muscular adaptations. The systematic review by Henselmans et al. (2022) [18] found no significant positive impact on strength performance in any short-term study and no significant positive effect on strength development in 16 out of 17 studies. In the sole study showing superior strength development in the higher-carbohydrate group, the lower-carbohydrate group had significantly lower energy intake and greater fat loss, suggesting that the lower strength development may be attributable to the lower energy intake [32]. In a systematic review and meta-analysis, Vargas-Molina et al. (2022) [33] investigated the ketogenic diet’s effect on fat-free mass increases during resistance training. They found no significant differences in fat-free mass changes between ketogenic and non-ketogenic studies. Another systematic review and meta-analysis in 2022 by Wang et al. [34] reported no significant differences in lean body mass or body composition between ketogenic and non-ketogenic diets in concurrent athletes.

However, prior studies, including the meta-analyses, may have been statistically underpowered to detect minor effects of carbohydrate or energy intake due to imperfect dietary compliance and high interindividual variability in training adaptations. Between-subject designs in exercise science suffer from major variance in muscle hypertrophy outcomes due to differences in the participants’ (epi)genetics, lifestyle, motivation, etc., and this variance cannot be adequately separated from the intervention effect [35]. While within-subject designs have their own limitations, crossover trials considerably enhance statistical power [36]. Therefore, to investigate whether additional carbohydrate–energy intake improves resistance training adaptations, we conducted a quasi-randomized, longitudinal, counterbalanced crossover trial to assess the effect of additional carbohydrate–energy supplementation on muscular strength performance and muscle hypertrophy in well-trained men. The within-subject design in a relatively homogenous sample with presumably ingrained training and nutrition habits should reduce variance in training adaptations. Based on prior research, we expected null effects on neuromuscular development but increased fat gain if dietary energy intake significantly increased due to the carbohydrate–energy supplementation.

## 2. Materials and Methods

### 2.1. Ethical Approval and Participant Demographics

This study was conducted with prior review and approval from the Auburn Institutional Review Board (IRB approval #: STUDY00000295; date of approval 31 January 2025) in accordance with the most recent revisions of the Declaration of Helsinki, and was retrospectively registered as a clinical trial (ClinicalTrials.gov Identifier: NCT07600138; approval date: 19 May 2026). Participants were recruited via convenience and snowball sampling across Auburn University’s campus, combined with a list of students who had previously expressed interest in participating in studies, to obtain the largest logistically feasible sample size of our target population. Following verbal and written consent, males from the local area were enrolled into the study if they (i) were aged 18–40 years old, (ii) had a body mass index (BMI) not exceeding 35 kg/m^2^, (iii) had a minimum of two years resistance training experience at a frequency of four days/week, (iv) had no known cardio-metabolic disease (e.g., clinical obesity, diabetes, hypertension, heart disease) or any condition contraindicating participation in resistance training, and (v) had no known allergy to whey protein supplements. Since our participants were resistance-trained men, clinical obesity was defined not based on BMI but on a body fat percentage over 30% in addition to the presence of at least one cardiometabolic pathology or organ dysfunction [37].

### 2.2. Study Design

This study utilized a within-participant, counterbalanced crossover design. Participants completed baseline testing (PRE) and were quasi-randomized by alternating allocation to the protein-only (PRO) or protein-with-added-carbohydrates (CHO) groups. They then consumed the assigned supplements for 8 weeks, completed a mid-point testing battery (MID), crossed over to the opposing group for another 8 weeks, and returned for a final testing battery (POST). Each testing battery included body composition (fat and fat-free mass), muscle size (muscle thicknesses and cross-sectional area), strength (squat 1RM and peak torque), and muscular endurance (fatigue index). All outcomes were assessed with concealed group allocation for both the participant and assessor (double-blinding).

### 2.3. Body Composition Testing

Upon arriving at the lab after an overnight fast and a minimum period of 72 h without vigorous physical activity, participants submitted a urine sample (~1.5 mL) for assessment of urine-specific gravity (USG) using a portable refractometer (ATAGO; Bellevue, WA, USA). If USG was above 1.025, participants were provided with ~16 fl oz of water before continuing testing to normalize hydration status. This was only necessary for one participant. Following USG assessments, participants proceeded to height (PRE only) and body mass assessments using a digital column scale (Seca 769; Hanover, MD, USA). Participants were then escorted to a dedicated testing area and asked to lie supine for at least 5 min before completing a full-body dual-energy X-ray absorptiometry (DXA) scan (Lunar Prodigy; GE Corporation, Fairfield, CT, USA) to assess bone-free lean tissue mass (LM) and fat mass (FM). Test–retest reliability was previously determined for LM, yielding an intraclass correlation coefficient (ICC) of 0.99 and a standard error of measurement (SEM) of 0.36 kg. Following DXA scans, participants were assessed for total body water (TBW), intracellular water content (ICW), and extracellular water content (ECW) using bioelectrical impedance spectroscopy. Electrodes were attached to participants’ skin according to the manufacturer’s instructions (ImpediMed SFB7; ImpediMed Inc., Carlsbad, CA, USA). Each electrode site was shaved and cleaned with rubbing alcohol before electrode attachment, and readings were obtained within five seconds after entry of participant demographics into the device’s digital interface.

### 2.4. Ultrasound

Following bioelectrical impedance spectroscopy, assessments of lateral thigh muscle thickness (vastus lateralis and vastus intermedius, noted as LT MT hereafter) and vastus lateralis cross-sectional area (VL CSA) were conducted using B-mode ultrasound (LOGIQ S7 Expert, GE Corporation, Fairfield, CT, USA) with a linear array transducer (3–12 MHz, GE Corporation, Fairfield, CT, USA). Each participant lay on their side, with their hips and knees bent to 90° as measured with a goniometer. The greater trochanter and lateral epicondyle of the femur were palpated and a mark was made at half the distance between the two using a cloth tape measure and Sharpie marker. Following this initial marking, an additional mark was placed 1 inch anteriorly and measurements of LT and MT were taken here. Participants then lay supine for measurements of LT and MT. The probe center was placed at the mark in the transverse plane. The ultrasound technician placed and oriented the probe while an assistant captured the image. All settings were kept consistent across participants (depth: 5.0 cm, frequency: 12 MHz, gain: 55 dB, dynamic range: 72). Following LT MT measurements, VL CSA was captured using panoramic ultrasound (Logic View, GE Corporation, Fairfield, CT, USA). A flexible, semi-rigid pad was placed around the thigh at the 50% length mark to be used as a guide. The technician positioned the probe and a panoramic image was taken while guiding the probe anteriorly across the thigh. Assessments of elbow flexors’ muscle thickness (biceps brachii and brachialis, denoted as EF MT hereafter) were conducted with the same settings as for LT MT. Each participant lay supine with their arm at their side and palm supinated. A mark was placed at 60% of the distance between the acromion process of the scapula and the crease of the elbow. The technician oriented the probe while another individual captured the image.

All measurements were taken on the dominant side of the body, except in the case of two individuals with previous musculoskeletal injuries on the dominant side that altered the morphology of the muscle. The same technician conducted all ultrasound measures at PRE, MID, and POST. The technician was blinded to participant group allocation and participants were instructed not to reveal any details about the supplement they were given to the technician during the study. A pilot study was conducted to assess the technician’s test–retest reliability. Intraclass correlation coefficients for all three measurements (LT MT, EF MT, VL CSA) were >0.98 in eleven pilot participants. Standard error of measurement values were 0.12 cm for LT MT, 0.5 cm^2^ for VL CSA, and 0.06 cm for EF MT.

Images were analyzed using ImageJ version 1.54g (NIH, Bethesda, MD, USA). Both measurements of muscle thickness were measured using the distance between the bone and the subcutaneous adipose tissue. Cross-sectional area was measured using the polygon tool by tracing the border of the vastus lateralis. The technician analyzing the images (D.R.T.) was blinded to the participant’s condition.

### 2.5. Performance Testing

Following ultrasound imaging, one-repetition-maximum (1RM) back squat, knee extension peak torque, and knee extension muscular endurance were determined for each participant. Barbell back squats were first performed with an eccentric action to 100° of knee flexion (0° of knee flexion being a straight leg). An adjustable box was used to ensure participants achieved the desired depth. Participants were instructed to use the touch-and-go method, in which they would “tap” the box at the bottom of each repetition rather than fully sit on it. Participants completed a standardized warm-up protocol consisting of one set of ten repetitions at 50% of the participant’s self-estimated 1RM, one set of five repetitions at 75% 1RM, and one set of one repetition at 90% of 1RM. Participants then began 1RM attempts, with each attempt separated by 3–5 min of rest. Participants were allowed five attempts to determine their true 1RM. Two participants did not complete 1RM testing at POST due to low back pain; hence, a total of 18 participants were included in the 1RM analysis.

Ten minutes following 1RM back squat attempts, participants completed isokinetic dynamometry to determine knee extensor peak torque and muscular endurance (BioDex System 4; Mirion Medical, Shirley, NY, USA). First, participants completed six maximal knee extension actions at a velocity of 90°/s. Peak torque was determined as the highest torque produced during these six contractions. Participants then rested for five minutes before performing 30 maximal knee extensions at a velocity of 180°/s. A fatigue index was calculated as the percentage decrease in the average peak torque produced during the first five contractions compared to the average peak torque produced during the final five contractions.

### 2.6. Supplement Intervention

Participants received either the protein or protein-plus-carbohydrate supplement after completing PRE testing by study staff who were not involved in testing (M.L.M). Following the MID testing, participants were given the other supplement. Supplements were provided to the participants in clear, unlabeled containers to maintain participant blinding. The PRO supplement consisted of a whey protein blend containing whey protein isolate, whey protein concentrate, and hydrolyzed whey protein (Gold Standard 100% Whey; Optimum Nutrition, Downers Grove, IL, USA). Participants were instructed to consume 39 g of the supplement daily, with each serving providing 30 g of protein, 4 g of carbohydrates, and 2 g of fat. The CHO supplement consisted of a mix of the same protein supplement used for PRO plus a maltodextrin supplement (Maltodextrin; Nutricost, Vineyard, UT, USA). Thirty-nine grams of the protein supplement were mixed with fifty-three grams of the maltodextrin supplement and participants were instructed to consume ninety-two grams of servings of the mixture each day. Each serving of the mixture contained 30 g of protein, 54 g of carbohydrates, and 2 g of fat. On days when participants performed resistance exercise, they were instructed to consume the supplement within one hour of completing the session. On all other days, participants were allowed to drink the supplement at any time of day. Participants were instructed to maintain their dietary habits throughout both study phases. Participants who routinely supplemented with creatine and/or caffeine were asked to continue to do so throughout the study. Participants were asked to refrain from taking additional ergogenic supplements during the study period. After completion of each supplementation period, adherence was measured by having participants report the number of servings remaining in the container. To avoid participants reporting 0 servings remaining out of social desirability, all containers had more servings than required and participants were informed of this but not of the exact surplus.

### 2.7. Resistance Training

Participants were instructed to continue their habitual resistance training regimen throughout the study periods and not to alter their weekly set training volume per muscle group. Participants were asked to keep detailed training logs throughout the study. Total volume load was calculated for the initial week following baseline testing and for the final week of each training period (weeks 7 and 15) as the sum of the per-set volume load (load used x repetitions performed), and fractional weekly set volumes for the elbow flexors and quadriceps were calculated as per Pelland et al. [38].

### 2.8. Nutrition Tracking

Four-day food logs were completed within one week before PRE, MID, and POST testing. Each food log consisted of two weekdays and two weekend days. The participant food logs were input into MyFitnessPal by or with direct oversight from a registered dietician to improve reporting accuracy. The data, which included the allocated supplement, are expressed as 4-day average energy intakes (kcal/d) and macronutrient intakes (g/d) of all available data. Incomplete daily records, such as days in which a participant forgot what they ate, were not included and the dietician could contact the participants in case of implausible food logs, but this did not end up being necessary.

### 2.9. Statistics

For the primary analyses, linear mixed models were fit with condition (CHO vs. PRO), period (first vs. second intervention in the crossover), and sequence (CHO first vs. PRO first) as fixed effects with change scores (midpoint—baseline or post—midpoint) of all outcomes as dependent variables (LM, FM, 1RM, peak torque, elbow flexor MT, thigh MT, vastus lateralis CSA), except for the fatigue index, which was analyzed as an absolute variable. Statistical inference for fixed effects was based on type III tests, with denominator degrees of freedom approximated using the Satterthwaite method. Within-subject correlation across the two periods was modeled using an unstructured repeated covariance matrix at the subject level, rather than random intercepts, to account for potential negative correlations in neuromuscular adaptations. Models were estimated using restricted maximum likelihood (REML). Estimated marginal means (±SE) of the effect of condition were reported along with their between-condition difference scores (CHO—PRO ± SE), i.e., effect sizes, and their 95% confidence intervals (CIs).

As secondary sensitivity analyses, ANCOVAs were run for the midpoint levels of all outcome variables using baseline scores as covariates. Equality of error variances was assessed using Levene’s Test. The first intervention’s effects may be considered the most conservative, as they reflect changes from an unconfounded baseline. In contrast, the second phase, in a crossover design with outcomes that do not wash out, such as hypertrophy and strength development, may be affected by the first intervention. However, neuromuscular adaptations in well-trained individuals over an eight-week period are expected to be limited and analyzing only the first phase does not offer the benefits of a within-subject design, in particular the higher statistical power, so the first-phase analysis was only conducted as a sensitivity test to rule out substantial confounding due to an order or period effect.

Assumptions of normality and homoscedasticity were checked by visual inspection of Q-Q plots and histograms of the between-condition difference scores of the outcome variables, along with Shapiro–Wilk tests. Missing data were handled using partial likelihood contributions in the primary analyses and listwise deletion in the ANCOVAs and *t*-tests. The only missing data for the dependent variables were two post-values for squat 1RMs due to back pain.

Between-condition differences in training volume, nutrient intakes, and supplement adherence were analyzed using paired-sample *t*-tests.

All tests were two-sided, and statistical significance was set at *α* = 0.05. No adjustments for multiple comparisons were made; instead, results were interpreted as exploratory with emphasis on effect sizes and their CIs rather than on dichotomous significance. The data were analyzed in IBM SPSS Statistics 26.

## 3. Results

### 3.1. Participants and Adverse Effects

**Figure 1:**
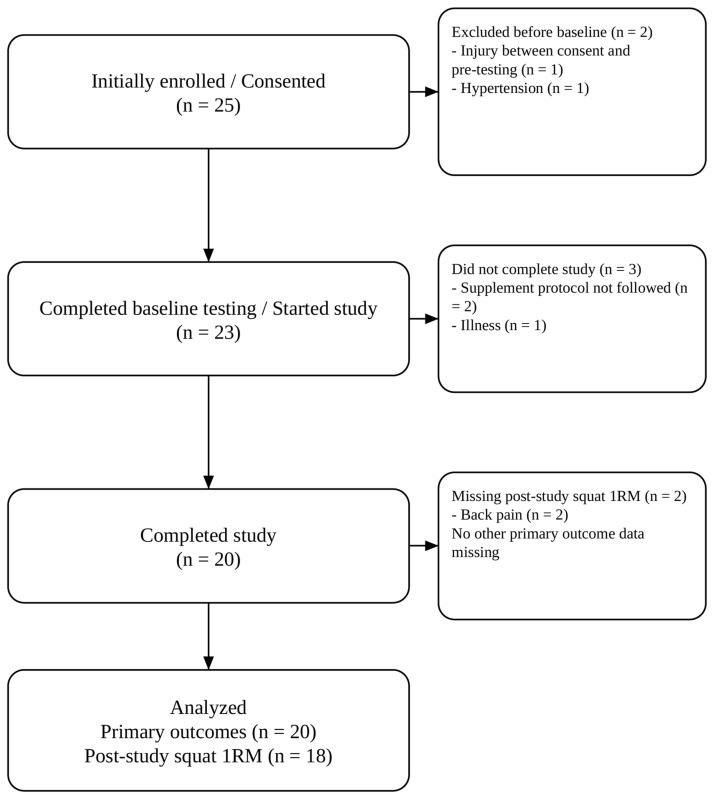
Participant flow diagram.

Twenty-five participants initially enrolled in the study. One participant was injured between consenting and pre-testing and thus could not participate. One participant was excluded due to hypertension. Two participants were excluded for not following the correct supplement protocol. Finally, one participant did not complete the study due to illness. In total, 20 participants completed the study (26.7 ± 4.9 years; BMI: 26.7 ± 2.7 kg/m^2^). Participants who completed the study reported having 9.7 ± 6.1 years of experience with resistance training, with an upper body training frequency of 3.1 ± 0.8 times per week, a lower body training frequency of 2.0 ± 0.6 times per week, and a baseline squat one-repetition-maximum (1RM)-to-body-weight ratio of 1.9 ± 0.4. Two participants could not complete post-study squat 1RMs due to back pain (one starting in the carbohydrate and one starting in the protein-only condition); otherwise, no data were missing for the primary outcomes. Figure 1 summarizes the participant flow.

### 3.2. Nutrition and Training Data

Dietary and training data are provided in Table 1. Total resistance training volume load was not significantly different between conditions (mean difference = 726 kg; 95% CI: −7074 to 8526 kg; *t*(16) = 0.20, *p* = 0.846) and weekly set volumes averaged to 14 for the quadriceps and 18 for the elbow flexors during both interventions. All participants reported training with high effort with the goal of becoming stronger and more muscular.

Supplement adherence was significantly higher in the CHO (99.67%) than the PRO condition (90.49%; *t*(19) = −6.77, *p* < 0.001). Daily energy intake was significantly higher during the CHO condition compared with the PRO condition (mean difference = 485 kcal/d; 95% CI: 99 to 871 kcal/d; *t*(15) = 2.68, *p* = 0.017). Carbohydrate intake was also significantly greater in the CHO condition (mean difference = 33 g/d; 95% CI: 1 to 65 g/d; *t*(15) = 2.21, *p* = 0.043). Protein intake did not differ between conditions (mean difference = 5 g/d; 95% CI: –22 to 12 g/d; *t*(15) = 0.61, *p* = 0.551), nor did fat intake (mean difference = 11 g/d; 95% CI: –27 to 6 g/d; *t*(15) = 1.40, *p* = 0.183). Note that the 485 kcal/d reported energy intake difference between conditions based on self-report and database entry did not equal the sum of reported macronutrient intakes based on standardized Atwater factors (251 kcal/d).

### 3.3. Data Assumptions

Visual inspection of Q-Q plots and histograms of the between-condition difference scores of the outcome variables revealed no notable abnormalities or heteroscedasticity. There were no physiologically implausible outliers. Shapiro–Wilk tests indicated approximately normal within-subject difference scores for all outcomes across conditions (all *p* ≥ 0.17), except for the fatigue index, which was borderline significant (*W* = 0.895, *p* = 0.048); this was not apparent in the plots. Levene’s Test showed no violations of the assumption of homogeneity of variances (all *p* ≥ 0.17).

### 3.4. Main Outcomes

**Figure 2:**
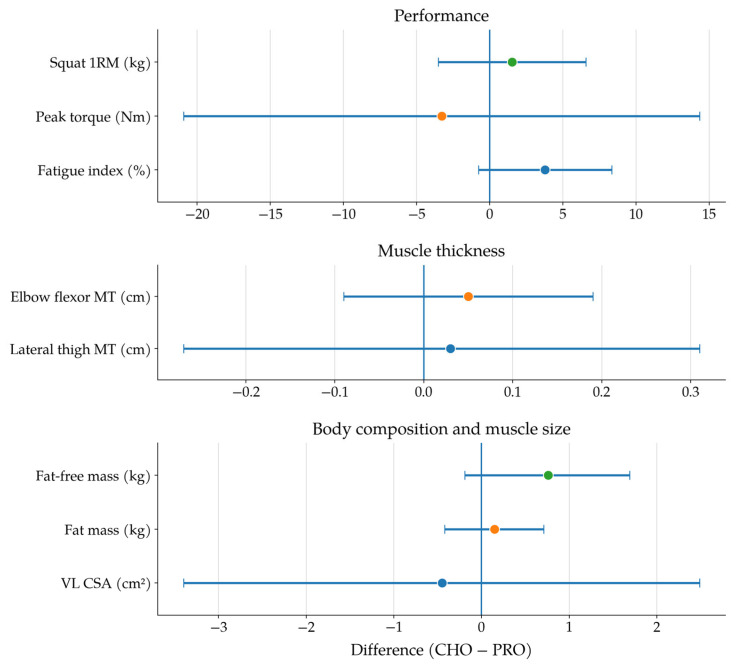
A multi-panel forest plot of the between-condition differences in estimated marginal means of the study outcomes ±95% CI. MT = muscle thickness. VL CSA = vastus lateralis cross-sectional area.

No outcome showed a statistically significant effect of condition (CHO vs. PRO) in the primary linear mixed models (all *p* ≥ 0.109). There was no significant period (all *p* ≥ 0.198, except for *p* = 0.062 for LM) or order effects (all *p* ≥ 0.219). The secondary between-subject ANCOVAs restricted to the midway point data, corrected for baseline variables, supported that there were no significant effects of condition on any outcome (all *p* ≥ 0.277), except for *p* = 0.095 for peak torque, representing a non-significant trend for greater changes in the PRO condition (see Supplementary File S1). Figure 2 summarizes the main results. Outcomes stratified by intervention sequence are presented in Supplementary File S1 Table S10.

#### 3.4.1. Performance Outcomes

There was no significant effect of condition on squat 1RM change: *F*(1, 18.286) = 0.402, *p* = 0.534. Estimated marginal means indicated similar increases under the PRO (5.24 ± 1.61 kg) and CHO (6.77 ± 1.63 kg) conditions, with an adjusted mean difference of 1.53 ± 2.41 kg (95% CI −3.52 to 6.57).

There was no significant effect of condition on knee extensor peak torque change: *F*(1, 18) = 0.153, *p* = 0.700. Estimated marginal means indicated similar changes under the PRO (−2.60 ± 4.91 Nm) and CHO (−5.89 ± 5.09 Nm) conditions, with an adjusted mean difference of −3.28 ± 8.40 Nm (95% CI −20.93 to 14.36).

There was no significant effect of condition on the fatigue index: *F*(1, 18) = 3.061, *p* = 0.097. Estimated marginal means indicated similar percentage loss in force output under the PRO (−38.61 ± 2.12) and CHO (−34.82 ± 2.17) conditions, with an adjusted mean difference of 3.79 ± 2.17 (95% CI −0.76 to 8.34).

#### 3.4.2. Body Composition Outcomes

There was no significant effect of condition on EF MT change: *F*(1, 18) = 0.511, *p* = 0.484. Estimated marginal means indicated similar changes under the PRO (−0.05 ± 0.05 cm) and CHO (0.03 ± 0.05 cm) conditions with an adjusted mean difference of 0.05 ± 0.07 cm (95% CI −0.09 to 0.19).

There was no significant effect of condition on LT MT change: *F*(1, 18) = 0.063, *p* = 0.805. Estimated marginal means indicated similar changes under the PRO (−0.05 ± 0.05 cm) and CHO (−0.02 ± 0.08 cm) conditions with an adjusted mean difference of 0.03 ± 0.13 cm (95% CI −0.27 to 0.31).

There was no significant effect of condition on VL CSA change: *F*(1, 18) = 0.104, *p* = 0.751. Estimated marginal means indicated similar increases under the PRO (1.82 ± 0.85 cm^2^) and CHO (1.36 ± 0.87 cm^2^) conditions with an adjusted mean difference of −0.45 ± 1.41 cm^2^ (95% CI −3.40 to 2.49).

There was no significant effect of condition on LM change: *F*(1, 18) = 2.848, *p* = 0.109. Estimated marginal means indicated similar changes under the PRO (−0.20 ± 0.33 kg) and CHO (0.55 ± 0.33 kg) conditions with an adjusted mean difference of 0.76 ± 0.45 kg (95% CI −0.19 to 1.69).

There was no significant effect of condition on FM change: *F*(1, 18) = 0.303, *p* = 0.588. Estimated marginal means indicated similar increases under the PRO (0.33 ± 0.24 kg) and CHO (0.48 ± 0.23 kg) conditions with an adjusted mean difference of 0.15 ± 0.27 kg (95% CI −0.42 to 0.71).

### 3.5. Sensitivity Analyses

In the sensitivity analyses restricted to the first intervention period, there were no significant effects of condition on body composition variables (LM (*F*(1, 17) = 1.262, *p* = 0.277, ηp^2^ = 0.069), FM *F*(1, 17) = 0.089, *p* = 0.769, ηp^2^ = 0.005). Likewise, no significant between-condition effects were observed for ultrasound-derived muscle size outcomes (LT MT: *F*(1, 17) = 1.088, *p* = 0.312, ηp^2^ = 0.060; EF MT: *F*(1, 17) = 0.211, *p* = 0.652, ηp^2^ = 0.012; VL CSA: *F*(1, 17) = 0.206, *p* = 0.656, ηp^2^ = 0.012). For performance outcomes, supplement condition did not significantly affect squat 1RM (*F*(1, 17) = 0.378, *p* = 0.547, ηp^2^ = 0.022) or knee extensor peak torque, though there was a non-significant trend favoring PRO (*F*(1, 17) = 3.134, *p* = 0.095, ηp^2^ = 0.156). The fatigue index also did not significantly differ between conditions (*F*(1, 18) = 0.048, *p* = 0.829, ηp^2^ = 0.003).

## 4. Discussion

We investigated the effect of supplemental carbohydrate–energy intake on resistance training adaptations in a counterbalanced, 8-week crossover design in male strength trainees. As intended, the carbohydrate–protein supplement resulted in significantly higher carbohydrate and energy but not fat or protein intakes than the protein-only supplement, although the achieved dietary contrast was 33 g instead of 50 g based on the participants’ food logs. The additional carbohydrate intake did not significantly improve any neuromuscular adaptations. The confidence intervals for all outcomes crossed zero and gains in vastus lateralis cross-sectional area and peak torque even directionally favored the protein-only condition. There was a non-significant trend towards greater strength-endurance in the carbohydrate-condition based on our fatigue index (*p* = 0.097), but this was not associated with significantly higher training volumes. Overall, our results are most consistent with a null effect of additional carbohydrate–energy intake on muscle hypertrophy and strength development within the context of well-trained men consuming a moderately higher carbohydrate intake for 8 weeks. Despite our design emphasis on statistical power, statistical detectability restricts how much non-significant results can be interpreted as true null effects. The wide confidence intervals do not rule out a (small) positive (or negative) effect of carbohydrates.

Our results are consistent with prior meta-analyses finding no significant effect of carbohydrate intake on training-induced fat-free mass increases [33,34] and a 2022 a systematic review by Henselmans et al. [18], which reported no significant difference in long-term strength development between high- and low-carbohydrate conditions in 15 out of 17 studies; one study favored the higher- and one the lower-carbohydrate condition.

A plausible explanation for the null findings is the limited glycogen depletion typically induced by conventional resistance training. Henselmans et al. [18] noted that training sessions involving ≤10 sets per muscle group generally result in glycogen reductions of ≤40%—a level usually insufficient to compromise neuromuscular performance, though higher depletion in type II fibers has been reported after higher training volumes. Mechanical tension is the primary stimulus for muscle hypertrophy, not acute metabolic stress or substrate availability [39], so when higher carbohydrate intakes do not result in higher training volumes, they should also not be expected to increase muscle hypertrophy.

Our results are also consistent with the lack of effect of higher energy intakes on resistance training adaptations in most studies, despite significantly higher rates of weight and fat gain in prior studies [9,10,11,12,13]. While energy deficiency may impair increases in fat-free mass [6], higher energy surpluses may not enhance muscle hypertrophy compared to energy-maintenance conditions. Based on thermodynamics, higher energy intakes should increase energy storage and some of this energy storage would plausibly occur in muscle tissue. In absolute terms, the increased carbohydrate–energy intake resulted in non-significantly higher fat and bone-free lean mass increases, but these were not associated with any consistent trend toward greater increases in our two strength performance measures or three muscle size measures (all *p*-values ≥ 0.484). Higher between-condition calorie differentials or study durations than we employed may be required to observe effects on body composition data. Given the unreliability of self-reported dietary data, we also cannot exclude the possibility of dietary compensation in energy intake to the extra supplemental energy intake. Greater fat-free mass increases would still not necessarily represent contractile tissue though, given the absence of energy intake’s effect on strength development in most studies, even in the context of energy deficit or large between-condition calorie differentials [6,7].

### Strengths and Limitations

The well-trained nature of our participants should support consistent training motivation and effort along with consistency in dietary and training variables over time, which was supported by non-significant differences between conditions in training volume and protein and fat intake, resulting in only carbohydrate and energy intake significantly differing between the conditions, as per design. Adherence to the protein-only supplement was significantly lower than to the carbohydrate supplement. While it is possible that differences in serving mass and palatability between supplements reduced the integrity of participant blinding and contributed to differential adherence, statistical significance in our adherence t-test was inflated by near absolute adherence (99.7%) and non-normality of data in the carbohydrate condition. The 9.2% lower adherence in the protein-only condition would be expected to reduce protein intake in the protein-only group by 2.8 g and energy intake by 14 kcal per day, and this difference should only magnify the dietary contrast between conditions, increasing the probability of finding between-condition effects. However, dietary intake was assessed via short-term self-reported food logs, which are prone to misreporting and may not fully capture compensation behaviors. Furthermore, dietary data on 4 participants, as well as volume-load for 3 participants, could not be obtained due to incomplete reporting. According to the reported carbohydrate intake, the achieved dietary contrast was 33 g instead of 50 g a day, suggesting that partial compensation in carbohydrate intake may have occurred in the carbohydrate condition. Moreover, energy intake was 2.5% higher and 6.9% lower in the carbohydrate and protein conditions, respectively, than predicted based on the reported macronutrients multiplied by standardized Atwater factors. Equivalence should not be expected though, because US food labels commonly use food-specific instead of standardized Atwater factors. Food-specific Atwater factors account for differences in net metabolizable energy resulting from a food’s amino acid and dietary fiber composition. Moreover, these discrepancies are within the variance found when comparing estimated energy intake from food logs in MyFitnessPal vs. another database [40].

Studying well-trained participants meant that further neuromuscular adaptations were unlikely to be large in magnitude, reducing statistical power, especially over a duration of 8 weeks. The sample homogeneity of trained men improved statistical power by reducing variance, but it also limits external validity to populations beyond trained men, including women, older adults, untrained individuals, athletes in considerable energy deficit, athletes with very low baseline carbohydrate intake and athletes performing high-volume glycogen-depleting training. Theoretically though, women are unlikely to benefit more from carbohydrate supplementation than men due to their more glucose-sparing metabolism [41].

Our crossover design improved statistical power to answer our research question. However, since muscle hypertrophy and strength adaptations do not wash out without extensive detraining (and even if washed out, may have stimulated muscle memory), it also introduced a considerable design limitation that the second intervention’s outcomes could be affected by the first, i.e., a carryover effect. Crossover designs are commonplace for nutritional interventions with resistance training (e.g., [42,43,44,45]), because the within-subject design has well-established benefits—majorly reduced variance in intervention outcomes and enhanced statistical power [35,36]—and carryover effects do not necessarily introduce bias. In our counterbalanced and quasi-randomized design, absolute carryover should cancel out and only differential carryover would introduce bias, though we cannot exclude this possibility [36]. Even in the case of asymmetric carryover, our design meets the conditions in which a crossover design can retain a power advantage over a parallel group design without inflating type I error, namely high within-subject correlations and same-sign treatment and carryover effects (i.e., carbohydrate intake is expected to be neutral to positive for our outcomes and so is the carryover due to lack of wash-out of muscle growth and strength development) [46]. Moreover, there is no established mechanism to our knowledge by which the differential carryover of our 8-week interventions would materially affect the effect of carbohydrate intake on neuromuscular adaptations in well-trained participants, though potential for bias remains. In line with theory, order and period effects were corrected for in the multivariate analysis and neither was significant for any outcome. The similar results in our sensitivity analysis of only the first intervention further support the robustness of the main findings. However, the statistical power of both the sensitivity analysis and the order and period effects limits the degree to which non-significance in these tests indicates true absence of a carryover effect. Overall, the crossover design represents both a design strength as well as a considerable limitation.

Further limitations of our design were that alternating allocation is not entirely random, there was no a priori power analysis and the trial was only retrospectively registered publicly on ClinicalTrials.gov, reducing transparency. However, the study design and all outcomes were specified before data collection, registered at Auburn University, and presented to all participants in the informed consent forms. The only change in the methodology that took place after data collection was that instead of an ANCOVA for the main analysis, we performed a mixed linear model for the main analysis with a first-period ANCOVA as a sensitivity analysis. This combination is more robust for our crossover design. The mixed linear model can properly model two correlated periods per person with correct standard errors and more calibrated small-sample inference, and it can handle the missing data points for the post-study squat 1RMs without requiring full listwise deletion of these participants, thereby enhancing statistical power. There was no statistical justification for an ANCOVA main analysis in our design beyond simplicity and familiarity.

Additional study strengths included measuring multiple outcomes and double-blinding of all outcomes. Due to the double-blinded measurements, we did not think to conduct a formal assessment of the blinding success. Multiple participants asked the study staff after the study which supplement they received when and reported not being able to tell which was which, suggesting blinding was successful, but we acknowledge that, in retrospect, differences in serving mass and palatability could have reduced blinding success.

Future research should consider pre-registered, well-controlled, longer-duration studies with adequately powered sample sizes and large between-condition differences in carbohydrate–energy intake to more precisely quantify if carbohydrate–energy intake affects resistance training adaptations.

## 5. Conclusions

In male resistance trainees, prescription of 50 g additional daily carbohydrate supplementation did not significantly improve measures of strength or muscle hypertrophy relative to protein-only supplementation across 8-week periods with habitual training and nutrition. Within the context of the achieved dietary contrast (33 g), these findings question the utility of modest increases in carbohydrate–energy intake outside of energy deficit to improve strength and hypertrophy outcomes in this population, but more high-powered research is warranted and our results should not be extrapolated to athletes involved in more glycogen-depleting exercise.

## Figures and Tables

**Table 1:** Dietary data, including supplementation and training volume. Values are mean ± SD. *: Significantly higher than in the protein condition.

	Carbohydrate Condition	Protein Condition
Carbohydrate intake (g/d)	248 ± 36 *	215 ± 49
Protein intake (g/d)	178 ± 47	173 ± 48
Fat intake (g/d)	109 ± 35	98 ± 34
Calorie intake (kcal/d)	2751 ± 645 *	2266 ± 672
Training volume load (kg)	42,657 ± 20,595	41,931 ± 21,496

## Data Availability

The original data presented in the study are openly available in the Open Science Framework at https://osf.io/enz8m/overview (accessed on 14 June 2026).

## Supplementary Materials

The following supporting information can be downloaded at: https://www.mdpi.com/article/10.3390/nu18121961/s1.
