# Supplementary material for: Effects of Modest Carbohydrate–Energy Supplementation on Resistance Training Adaptations in Trained Men: A Crossover Trial
Source: Nutrients. 2026 Jun 17;18(12):1961. doi: 10.3390/nu18121961 (PMC13305566; doi:10.3390/nu18121961)
Supplement: Supplementary file 1 [file nutrients-18-01961-s001.zip › nutrients-4347189-supplementary.pdf]

Supplementary File S1: Secondary Analysis Using Period 1 Only and Outcomes by Intervention Sequence

Manuscript: Henselmans, M.; Tiede, D.R.; Plotkin, D.L.; Mattingly, M.L.; Harbour, E.R.; Anglin, D.A.; Fruge, A.D.; Vårvik, F.T.; Roberts, M.D.; Izquierdo, M. *The Effect of Carbohydrate–Energy Intake on Neuromuscular Development: A Cross-Over Trial. Nutrients* 2026.

These analyses correspond to the sensitivity analyses reported in Section 3.5 of the main manuscript. To corroborate the primary linear mixed-model analyses, the effect of supplementation condition on each outcome was re-estimated using only data from the first 8-week intervention period (i.e., excluding the second phase of the cross-over). For all outcomes except the fatigue index, a one-way between-subjects ANCOVA was used with the corresponding baseline (PRE) value as a covariate. Because the fatigue index was not measured at baseline, it was analyzed with a one-way ANOVA. Levene's test of equality of error variances was inspected for each outcome and was non-significant throughout (all $p > .05$).

All analyses were conducted in IBM SPSS Statistics 26 with $\alpha = .05$.

Table S1. Summary of period-1-only sensitivity analyses for the effect of supplementation (carbohydrate-protein vs. protein-only) on neuromuscular and body-composition outcomes (N = 20; n = 10 per condition).

**Outcome**	***F***	***df***	***p***	η^2_p	**Levene's *F***	**Levene's *p***
Lean mass (kg)	1.262	(1, 17)	.277	0.069	1.214	.285
Fat mass (kg)	0.089	(1, 17)	.769	0.005	1.868	.188
Lateral thigh muscle thickness (cm)	1.088	(1, 17)	.312	0.060	0.315	.582
Elbow flexor muscle thickness (cm)	0.211	(1, 17)	.652	0.012	0.079	.781
Vastus lateralis cross-sectional area (cm^2)	0.206	(1, 17)	.656	0.012	1.467	.242
Back squat 1RM (kg)	0.378	(1, 17)	.547	0.022	0.566	.462
Knee extensor peak torque (Nm)	3.134	(1, 17)	.095	0.156	0.914	.352
Fatigue index (%)	0.048	(1, 18)	.829	0.003	0.262	.615

Note. Each row reports the effect of the supplement condition (CHO vs. PRO) in a separate one-way between-subjects ANCOVA on the period-1 (8-week) outcome with the corresponding baseline value as a covariate, except for the fatigue index, which was analyzed by one-way ANOVA (no baseline measurement available). η^2_p = partial eta-squared, computed as *SS* effect / (*SS* effect + *SS* error).

Detailed ANCOVA Output by Outcome

The tables below reproduce the full SPSS Tests of Between-Subjects Effects output for each outcome, along with Levene's test of equality of error variances. Sums of squares are Type III. Sample size is N = 20 (n = 10 per condition) in all analyses.

Table S2. ANCOVA on Lean mass (kg). Covariate: baseline lean mass.

Source	Type III SS	*df*	MS	*F*	*p*
Corrected model	906.377	2	453.188	362.341	<.001
Intercept	15.617	1	15.617	12.487	.003
Baseline lean mass	883.671	1	883.671	706.528	<.001
**Supplement**	**1.578**	**1**	**1.578**	**1.262**	**.277**
Error	21.262	17	1.251		
Total	94,289.652	20			
Corrected total	927.639	19			

Note. R^2 = .977 (adjusted R^2 = .974). Levene's test: $F(1, 18) = 1.214$, $p = .285$.

Table S3. ANCOVA on Fat mass (kg). Covariate: baseline fat mass.

Source	Type III SS	*df*	MS	*F*	*p*
Corrected model	1,016.018	2	508.009	504.068	<.001
Intercept	0.001	1	0.001	0.001	.976
Baseline fat mass	953.396	1	953.396	945.999	<.001
**Supplement**	**0.090**	**1**	**0.090**	**0.089**	**.769**
Error	17.133	17	1.008		
Total	7,562.565	20			
Corrected total	1,033.151	19			

Note. R^2 = .983 (adjusted R^2 = .981). Levene's test: $F(1, 18) = 1.868$, $p = .188$.

Table S4. ANCOVA on Lateral thigh muscle thickness (cm). Covariate: baseline LT muscle thickness.

Source	Type III SS	*df*	MS	*F*	*p*
Corrected model	7.623	2	3.812	27.327	<.001
Intercept	0.518	1	0.518	3.712	.071
Baseline LT muscle thickness	6.686	1	6.686	47.934	<.001
**Supplement**	**0.152**	**1**	**0.152**	**1.088**	**.312**
Error	2.371	17	0.139		
Total	596.547	20			
Corrected total	9.994	19			

Note. R^2 = .763 (adjusted R^2 = .735). Levene's test: $F(1, 18) = 0.315$, $p = .582$.

Table S5. ANCOVA on Elbow flexor muscle thickness (cm). Covariate: baseline EF muscle thickness.

Source	Type III SS	*df*	MS	*F*	*p*
Corrected model	0.978	2	0.489	8.584	.003
Intercept	0.160	1	0.160	2.814	.112
Baseline EF muscle thickness	0.840	1	0.840	14.750	.001
**Supplement**	**0.012**	**1**	**0.012**	**0.211**	**.652**
Error	0.969	17	0.057		
Total	317.324	20			
Corrected total	1.947	19			

Note. R^2 = .502 (adjusted R^2 = .444). Levene's test: $F(1, 18) = 0.079$, $p = .781$.

Table S6. ANCOVA on Vastus lateralis cross-sectional area (cm^2). Covariate: baseline VL CSA.

Source	Type III SS	*df*	MS	*F*	*p*
Corrected model	427.987	2	213.994	11.725	.001
Intercept	45.684	1	45.684	2.503	.132
Baseline VL CSA	396.737	1	396.737	21.738	<.001
**Supplement**	**3.755**	**1**	**3.755**	**0.206**	**.656**
Error	310.261	17	18.251		
Total	28,773.320	20			
Corrected total	738.248	19			

Note. R^2 = .580 (adjusted R^2 = .530). Levene's test: $F(1, 18) = 1.467$, $p = .242$.

Table S7. ANCOVA on Back squat 1RM (kg). Covariate: baseline 1RM.

Source	Type III SS	*df*	MS	*F*	*p*
Corrected model	21,372.673	2	10,686.337	212.234	<.001
Intercept	535.239	1	535.239	10.630	.005
Baseline 1RM	21,322.238	1	21,322.238	423.465	<.001
**Supplement**	**19.025**	**1**	**19.025**	**0.378**	**.547**
Error	855.980	17	50.352		
Total	646,502.699	20			
Corrected total	22,228.654	19			

Note. R^2 = .961 (adjusted R^2 = .957). Levene's test: $F(1, 18) = 0.566$, $p = .462$.

Table S8. ANCOVA on Knee extensor peak torque (Nm). Covariate: baseline peak torque.

Source	Type III SS	*df*	MS	*F*	*p*
Corrected model	30,105.311	2	15,052.656	27.135	<.001
Intercept	63.703	1	63.703	0.115	.739
Baseline peak torque	29,387.709	1	29,387.709	52.976	<.001
**Supplement**	**1,738.726**	**1**	**1,738.726**	**3.134**	**.095**
Error	9,430.597	17	554.741		
Total	1,433,033.540	20			
Corrected total	39,535.908	19			

Note. R^2 = .761 (adjusted R^2 = .733). Levene's test: $F(1, 18) = 0.914$, $p = .352$.

Table S9. ANCOVA on Fatigue index (%). One-way ANOVA (no covariate; baseline not measured).

Source	Type III SS	*df*	MS	*F*	*p*
Corrected model	5.471	1	5.471	0.048	.829
Intercept	24,680.933	1	24,680.933	217.881	<.001
**Supplement**	**5.471**	**1**	**5.471**	**0.048**	**.829**
Error	2,038.991	18	113.277		
Total	26,725.395	20			
Corrected total	2,044.462	19			

Note. R^2 = .003 (adjusted R^2 = -.053). Levene's test: $F(1, 18) = 0.262$, $p = .615$.

Table S10. Outcomes by treatment sequence.

Outcome	Sequence	PRE (baseline)	MID (wk 8)	POST (wk 16)
**Lean mass (kg)**	CHO-first	66.8 ± 7.1	67.3 ± 6.5	67.3 ± 6.2
	PRO-first	68.6 ± 8.6	69.4 ± 7.6	68.9 ± 8.1
**Fat mass (kg)**	CHO-first	16.2 ± 8.7	16.3 ± 8.9	17.1 ± 9.2
	PRO-first	19.5 ± 5.2	19.8 ± 5.3	20.1 ± 5.4
**Lateral thigh muscle thickness (VL+VI, cm)**	CHO-first	5.23 ± 0.89	5.20 ± 0.86	5.12 ± 0.81
	PRO-first	5.56 ± 0.65	5.63 ± 0.51	5.52 ± 0.45
**Vastus lateralis cross-sectional area (cm^2)**	CHO-first	33.9 ± 5.6	36.2 ± 6.6	37.6 ± 6.9
	PRO-first	36.0 ± 6.2	38.7 ± 5.9	38.7 ± 5.7
**Elbow flexor muscle thickness (cm)**	CHO-first	4.07 ± 0.36	4.05 ± 0.29	4.14 ± 0.30
	PRO-first	3.90 ± 0.26	3.89 ± 0.34	3.86 ± 0.34
**Back squat 1RM (kg)**	CHO-first	169.9 ± 37.0	178.3 ± 37.7	180.5 ± 38.9
	PRO-first	168.5 ± 39.5	175.1 ± 32.2	177.8 ± 34.8
**Knee extensor peak torque (90°/s, Nm)**	CHO-first	251.9 ± 45.7	258.0 ± 42.9	250.2 ± 45.1
	PRO-first	286.3 ± 40.4	269.9 ± 49.7	270.6 ± 48.1
**Fatigue index (%)**	CHO-first	-34.1 ± 11.4	-35.7 ± 9.5	-35.9 ± 8.1
	PRO-first	-36.5 ± 12.7	-34.6 ± 11.7	-40.1 ± 9.5
**Body weight (kg)**	CHO-first	85.8 ± 13.1	82.4 ± 11.4	83.2 ± 11.6
	PRO-first	91.3 ± 7.2	92.5 ± 6.1	92.1 ± 6.7
**Training volume load (lb)**	CHO-first	81,298 ± 35,064	94,109 ± 48,838	89,264 ± 41,268
	PRO-first	75,352 ± 40,233	96,018 ± 56,212	93,969 ± 44,570
**Energy intake (kcal/d)**	CHO-first	2,657 ± 604	2,427 ± 372	1,959 ± 655
	PRO-first	2,689 ± 419	2,661 ± 485	3,169 ± 703
**Protein intake (g/d)**	CHO-first	177.9 ± 45.5	162.8 ± 43.6	170.7 ± 47.2
	PRO-first	161.4 ± 51.2	175.9 ± 52.4	197.4 ± 46.9
**Carbohydrate intake (g/d)**	CHO-first	227.7 ± 68.4	245.5 ± 30.1	187.0 ± 39.4
	PRO-first	236.3 ± 53.8	250.0 ± 37.0	250.3 ± 44.2
**Fat intake (g/d)**	CHO-first	109.8 ± 35.7	97.4 ± 27.3	84.7 ± 26.1
	PRO-first	128.8 ± 33.3	115.0 ± 36.7	123.0 ± 41.2
